# Transcatheter Closure of Left Ventricular Outflow Tract–to–Left Atrium Fistula

**DOI:** 10.1016/j.jaccas.2021.02.040

**Published:** 2021-05-19

**Authors:** Yasser Sammour, Sanchit Chawla, Rayji S. Tsutsui, Jayendrakumar Patel, Serge Harb, Samir Kapadia

**Affiliations:** Heart and Vascular Institute, Cleveland Clinic Foundation, Cleveland, Ohio, USA

**Keywords:** Amplatzer Vascular Plug II, intracardiac fistula, transcatheter closure, AVP II, Amplatzer Vascular Plug II, LA, left atrium, LVOT, left ventricular outflow tract, SAVR, surgical aortic valve replacement, TAVR, transcatheter aortic valve replacement, TEE, transesophageal echocardiography

## Abstract

Surgical and rarely transcatheter aortic valve replacement can be complicated by intracardiac fistula. Transcatheter closure of those shunts has been previously reported with favorable results. We describe a case of percutaneous closure of left ventricular outflow tract–to–left atrium fistula after surgical aortic valve replacement using an Amplatzer Vascular Plug II. (**Level of Difficulty: Advanced.**)

## Central Illustration

**Figure:**
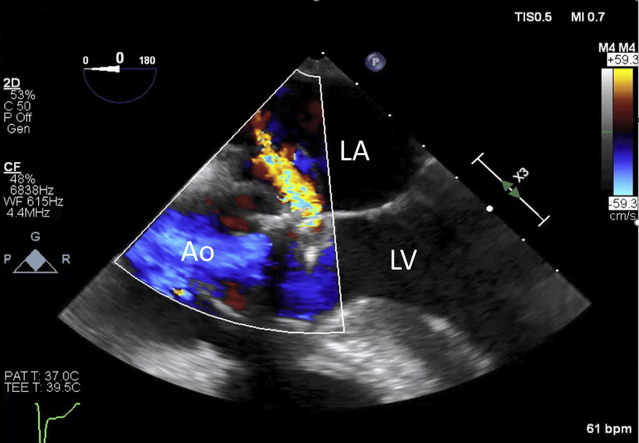

## History of Presentation

An 83-year-old Caucasian man presented to our institution for spinal surgery. The post-operative course was complicated by acute worsening of anemia that was present at baseline prior to surgery, with a decrease of hemoglobin from 10 to 5.7 g/dl on post-operative day 1, which persisted despite repeated blood transfusion.

**Learning Objectives**

• To emphasize the utility of percutaneous intracardiac fistula closure as a viable alternative, especially for poor surgical candidates.
• To demonstrate that the Amplatzer Vascular Plug II is softer compared with other devices and thus can be associated with less post-procedural hemolysis.

## Medical History

The patient had a history of heart failure with preserved ejection fraction, coronary artery disease, peripheral artery disease, hypertension, hyperlipidemia, chronic kidney disease, bicuspid aortic valve, and severe aortic stenosis. He had recently undergone surgical aortic valve replacement (SAVR) at an outside hospital 7 months prior to presentation with initially a 25-mm Trifecta valve (Abbott Cardiovascular, Plymouth, Minnesota), followed by redo SAVR with a 27-mm Magna Ease valve (Edwards Lifesciences, Irvine, California) because of moderate aortic regurgitation.

## Differential Diagnosis

The differential diagnosis included hemolytic anemia, hemorrhagic anemia, and anemia of chronic disease.

## Investigations

**Figure 1:**
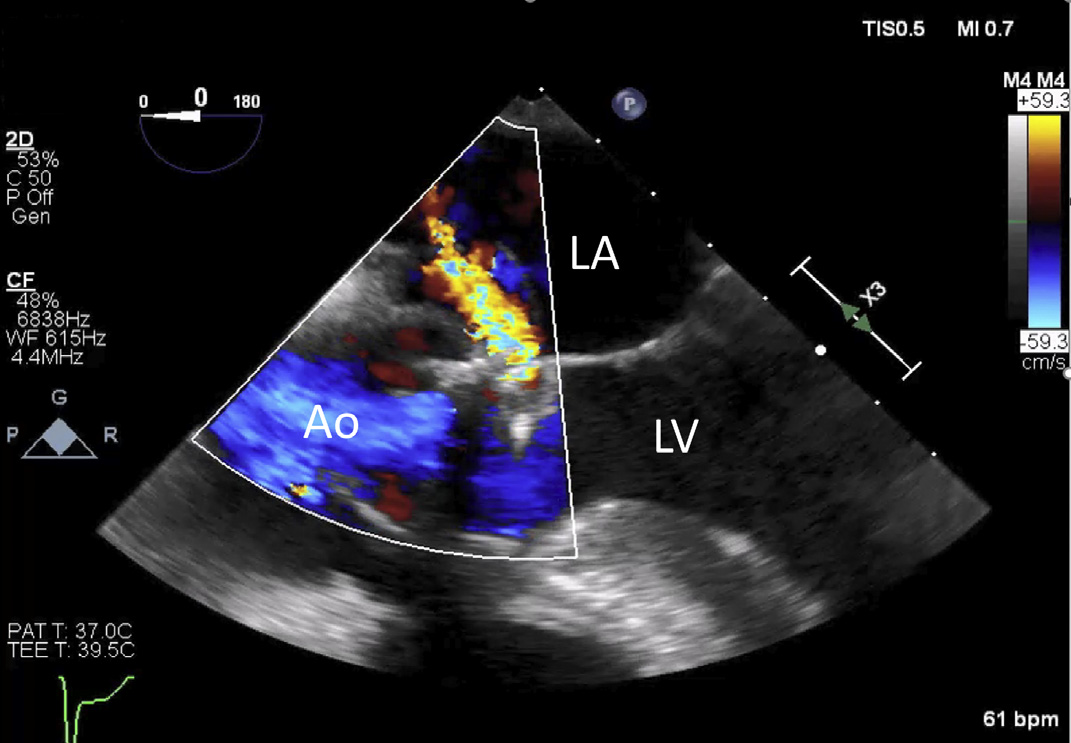
Transesophageal Echocardiography With Color Doppler Showing Left Ventricular Outflow Tract-to-LA Fistula Ao =aorta; LA = left atrium; LV = left ventricle.

A small right retroperitoneal hematoma was seen on computed tomography. Fecal occult blood test results were negative. Hemolytic work-up was notable for lactate dehydrogenase 517 U/l and low haptoglobin (<10 mg/dl). Transthoracic echocardiography showed a left ventricular ejection fraction of 63% and turbulent high-velocity systolic flow from the inferior aspect of the aortic prosthesis to the left atrium (LA) consistent with left ventricular outflow tract (LVOT)–to–LA fistula. Transesophageal echocardiography (TEE) confirmed a large pulsatile echolucency adjacent to the prosthetic valve with associated LVOT-to-LA fistula (Figure 1, Video 1). Upon review of previous echocardiograms, perivalvular systolic flow was detected in the echocardiogram obtained after the cardiopulmonary bypass of the second SAVR, leading to a diagnosis of acquired LVOT-to-LA fistula and secondary hemolysis. Because of concern for endocarditis in the setting of recent SAVR, a thorough infectious work-up was done that showed negative blood cultures and fungal battery and normal results on whole-body ^18^F-fluorodeoxyglucose positron emission tomography/computed tomography.

## Management

The patient was evaluated by the interventional cardiology team and was taken to the catheterization laboratory for common femoral vein using a modified Seldinger technique.

Through the 9-F sheath in the left common femoral vein, the AcuNAV intracardiac echocardiographic probe (Siemens Healthineers, Erlangen, Germany) was advanced to the right atrium. Transseptal puncture was done using a Brockenbrough needle and electrocautery under intracardiac echocardiographic guidance. A Mullins sheath was then advanced to cross the septum. Left atrial pressure was normal. Next, we advanced a 230-cm Toray guidewire into the left atrium and removed the Mullins sheath. The interatrial septum was then dilated using a 14-F Inoue dilator. A small-curve 8.5-F Agilis-NxT steerable sheath (Abbott Cardiovascular) was passed into the LA.

After that, we crossed the aortic valve using a 0.035-inch 260-cm Straight Fixed Core wire and advanced the Amplatz left AL1 diagnostic catheter into the left ventricle. The Straight wire was then swapped out for a 0.035-inch Amplatz Extra Stiff wire (Cook Medical, Bloomington, Indiana). We upsized the 5-F sheath in the right common femoral artery to an 8-F sheath. We then advanced a 6-F 90-cm Shuttle Guiding Sheath (Cook Medical) into the left ventricle, through which we inserted a 5-F internal mammary artery diagnostic catheter.

**Figure 2:**
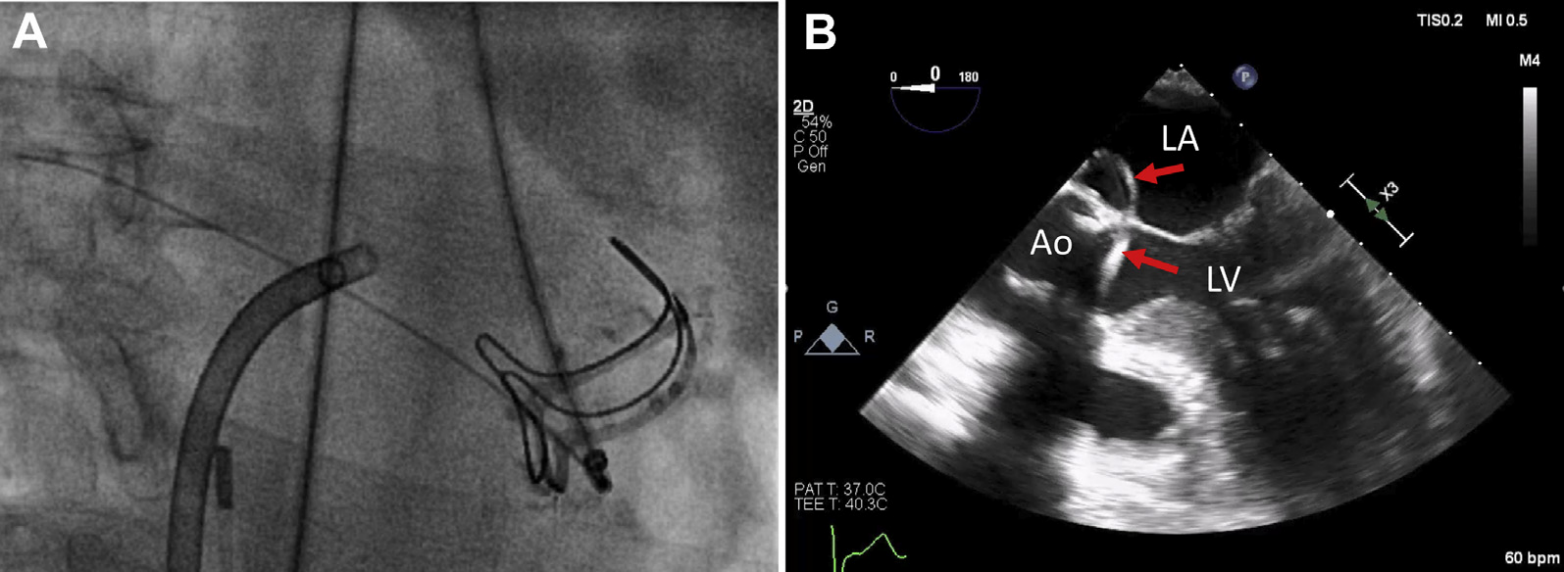
Crossing the Fistula **(A)** The fistula was crossed from the left ventricular outflow tract into the left atrium and pulmonary vein using a stiff-angled Glidewire under fluoroscopic guidance. **(B)** The Glidewire **(red arrows)** can be seen crossing the defect on transesophageal echocardiography. Abbreviations as in Figure 1.

**Figure 3:**
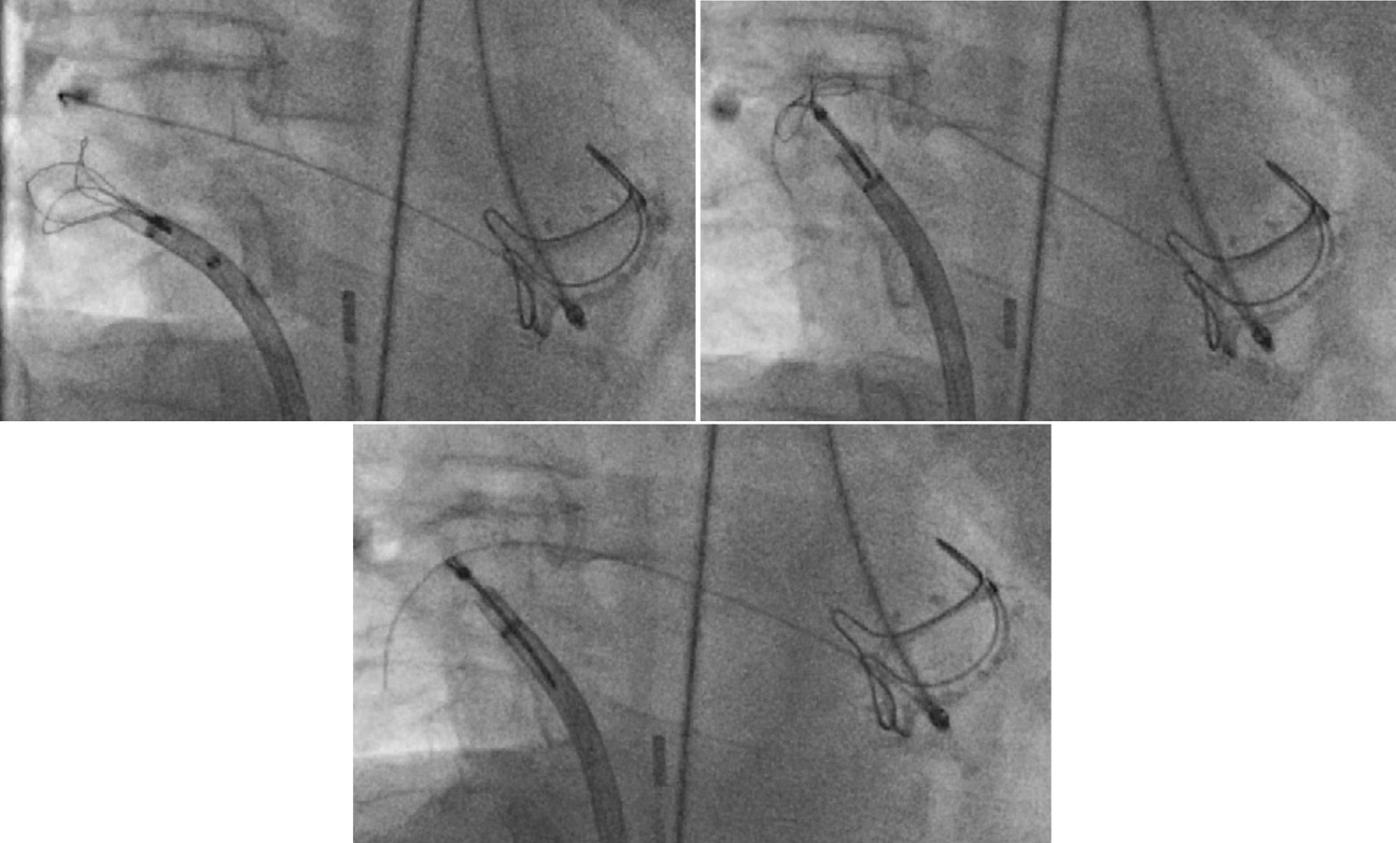
En-Snare Device Was Used to Capture the Stiff-Angled Glidewire and Externalize It Through the Agilis-NxT Steerable Sheath in the Left Atrium

**Figure 4:**
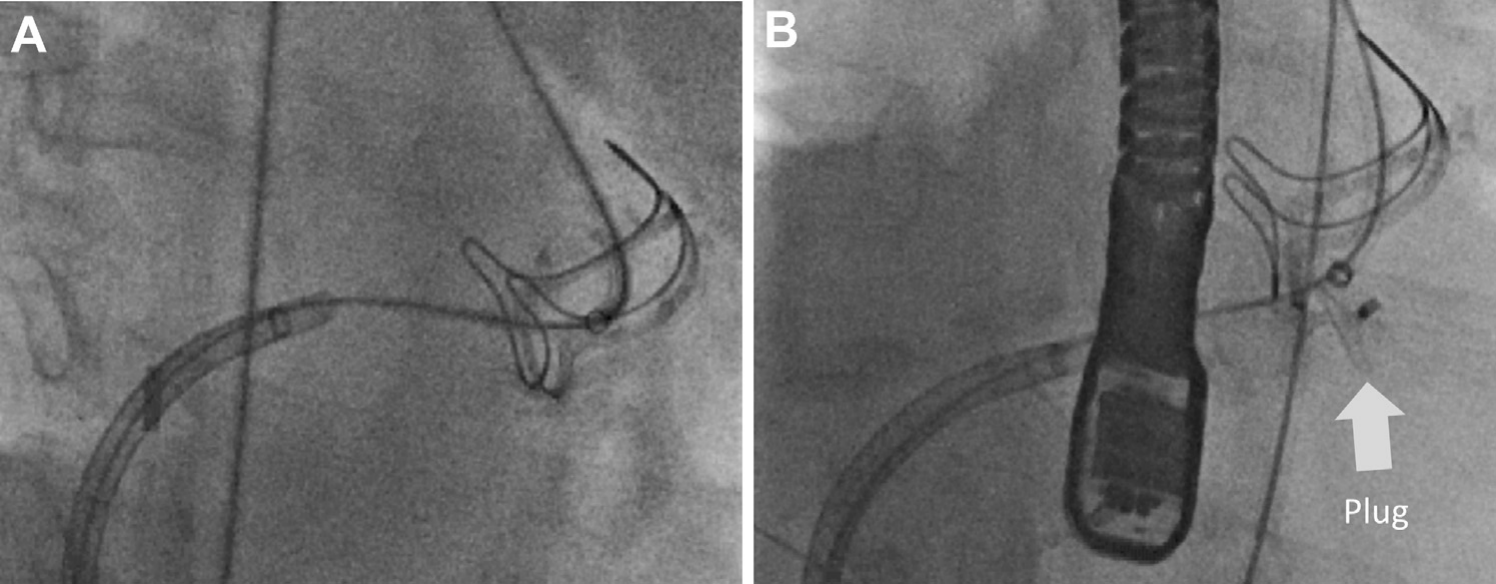
Advancement of the Amplatzer Vascular Plug II **(A)** The Glidewire was used as a rail to advance the Shuttle Guiding Sheath through the defect into the left atrium. **(B)** The Amplatzer Vascular Plug II was advanced into the fistula.

**Figure 5:**
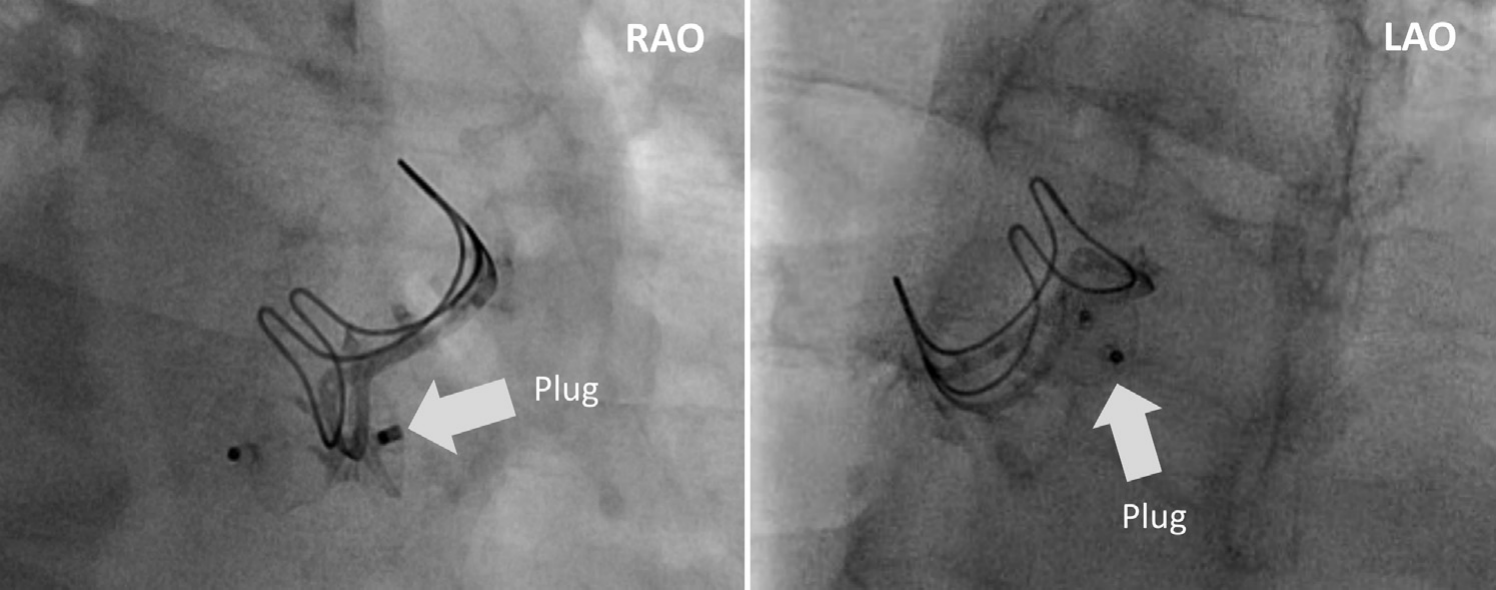
The Released Amplatzer Vascular Plug II Can Be Seen in RAO and LAO Projections LAO = left anterior oblique; RAO = right anterior oblique.

**Figure 6:**
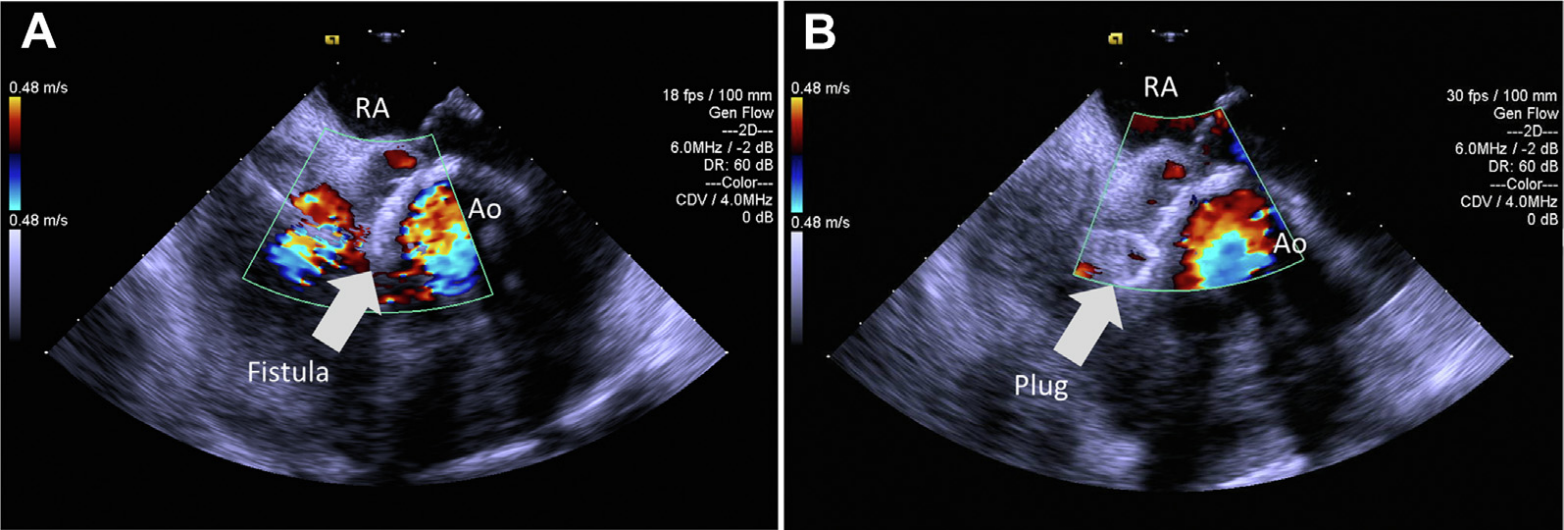
Intracardiac Echocardiography With Color Doppler Showing LVOT-to-LA Fistula Before and After Amplatzer Vascular Plug II Release **(A)** Before and **(B)** after Amplatzer Vascular Plug II release. Abbreviations as in Figure 1.

**Figure 7:**
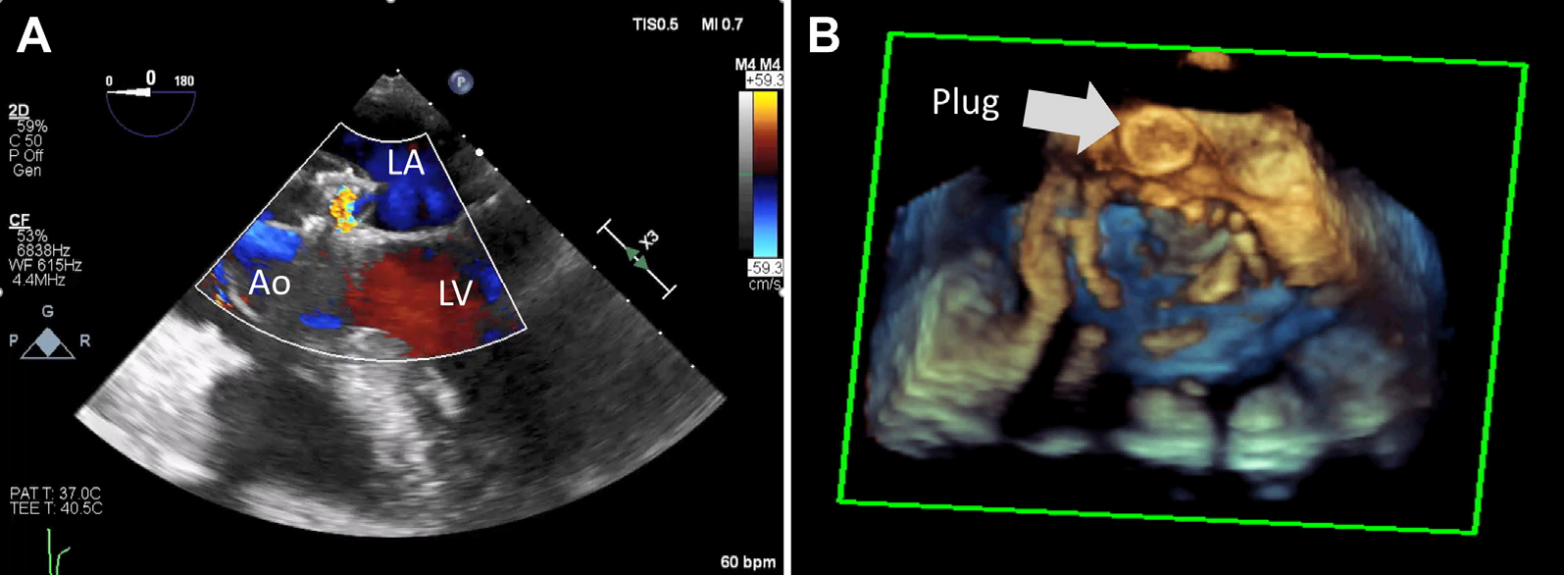
Post-Procedural Changes on Transesophageal Echocardiography **(A)** Transesophageal echocardiography with color Doppler showing minimal communication between the left ventricular outflow tract and LA. **(B)** The released Amplatzer Vascular Plug II can be seen on 3-dimensional transesophageal echocardiography with live multiplanar reconstruction. Abbreviations as in Figure 1.

Thereafter, under fluoroscopic guidance, we crossed the fistula from the LVOT into the LA and pulmonary vein using a stiff-angled Glidewire (Terumo, Tokyo, Japan) (Figure 2). Following that, a 9- to 15-mm En-Snare device (Merit Medical, South Jordan, Utah) was used to capture the stiff-angled Glidewire and externalize it through the Agilis-NxT steerable sheath in the LA (Figure 3, Video 2). The Glidewire was used as a rail to advance the 6-F Shuttle Guiding Sheath through the defect into the LA (Figure 4A). Next, we advanced a 12-mm Amplatzer Vascular Plug II (AVP II, Abbott Cardiovascular) into the fistula (Figure 4B). The 8-mm plug was initially tried but could not completely ameliorate the flow. We confirmed appropriate positioning and stability using fluoroscopy, intracardiac echocardiography, and TEE and then released the plug (Figures 5, 6 and 7, Video 3). TEE showed minimal communication between the LVOT and LA, with improved hemodynamic status and without any prosthetic valve dysfunction, new mitral regurgitation, or pericardial effusion (Video 4). The right femoral arteriotomy site was closed with an Angio-Seal device (Terumo), and venous access sites were manually compressed.

## Discussion

We describe a case of iatrogenic LVOT-to-LA fistula, secondary to SAVR, that presented with chronic hemolytic anemia and was percutaneously closed using AVP II.

Iatrogenic intracardiac shunts can occur after SAVR as a result of direct mechanical injury (1) or, less commonly, after transcatheter aortic valve replacement (TAVR) with compression of annular calcium during valve expansion or direct mechanical trauma by the delivery system, including the introducer (2). Severely calcified annulus, prosthesis oversizing, aortic root enlargement, and deeper valve implantation can potentially increase the risk for this complication (1). Prosthetic valve endocarditis can also be a cause (3). In our patient, the bicuspid aortic valve anatomy, and the need for 2 SAVRs were likely the cause.

The clinical presentation may vary depending on fistula location and/or size. The majority of patients remain asymptomatic, but approximately one-third may develop heart failure symptoms due to significant shunting (2). Our patient presented with chronic hemolytic anemia that improved after fistula repair.

Conservative management may be appropriate in asymptomatic patients. However, patients with worsening heart failure symptoms should be considered for closure (2). Surgical correction is usually the gold standard, but enhanced operator experience with transcatheter approaches has promoted new techniques and devices for minimally invasive closure, especially given that many of these patients are poor surgical candidates (1).

Transcatheter closures using septal occluders, ductal occluders, and vascular plugs have been previously reported with satisfactory results including shorter recovery time and lower morbidity (4). The AVP II is a self-expanding device consisting of a densely braided multilayer Nitinol mesh with 3 disks of equal diameter that shorten by compression and adjust to variable landing zones, thus allowing better sealing (5). In our case, we introduced the distal disk of the plug into the LA and maintained the proximal disk in the aorta, while the central disk expanded in between to seal the defect. We believe that the AVP II is softer and causes less hemolysis compared with other devices, which are relatively stiffer. Saxon et al. (6) also described percutaneous closure of aorto–right ventricular fistula after TAVR using the AVP II. They recommended that optimal plug diameter should be 50% above the defect maximal diameter. If endocarditis is suspected as a possible etiology for the fistula (3), it is advisable to delay placement of closure devices until adequate treatment of endocarditis is ensured, or ideally surgery should be considered. Potential complications for Amplatzer devices include perforation, interference with the hemodynamic status of mechanical valves, coronary artery obstruction, and device embolization if not properly sized or placed.

## Follow-Up

The following day, the patient had persistent oozing and bleeding from the access site that required injection with lidocaine and epinephrine. The next day, hemoglobin decreased to 6.4 g/dl, and the patient was given 1 U of blood, with mild improvement in hemoglobin; thus an additional 2 U was given, with improved hemoglobin to 9.6 g/dl. No further transfusion was required during the rest of the hospitalization. Hemolytic markers continued to improve, with lactate dehydrogenase of 278 U/l and haptoglobin of 64 mg/dl on discharge. Repeat transthoracic echocardiography done 1 month later showed no obvious residual high-velocity flow into the LA.

## Conclusions

SAVR and less commonly TAVR can be complicated by intracardiac shunts. Transcatheter closure using the AVP II is potentially safe and feasible. However, it should be reserved for patients with experienced heart team evaluation.

## Funding Support and Author Disclosures

The authors have reported that they have no relationships relevant to the contents of this paper to disclose.

## Appendix

Supplemental Video 1 Transesophageal Echocardiography With Color Doppler Showing LVOT-to-LA Fistula.

Supplemental Video 2 Use of En-Snare Device to Capture the Stiff-Angled Glidewire and Externalize It Through the Agilis-NxT Steerable Sheath in the Left Atrium.

Supplemental Video 3 The Amplatzer Vascular Plug II Was Released After Confirmation of Positioning Using Transesophageal Echocardiography and Intracardiac Echocardiography.

Supplemental Video 4 Transesophageal Echocardiography With Color Doppler Showing Minimal Communication Between The Left Ventricular Outflow Tract and the Left Atrium.
